# Campylobacter rectus, an Uncommon Isolate of Multiple Splenic Abscesses, in a 79-Year-Old Chinese Woman With an Attempt at Conservative Management Followed by Operative Intervention: A Case Report

**DOI:** 10.7759/cureus.51594

**Published:** 2024-01-03

**Authors:** Christian Przeslawski, Alise Haddad, Alona Salita, Michel Hanna, Jonathan Lezotte

**Affiliations:** 1 General Surgery, Corewell Health Southeast - Farmington Hills Campus, Farmington Hills, USA; 2 Infectious Disease, Oakland University William Beaumont School of Medicine, Auburn Hills, USA; 3 Infectious Disease, Corewell Health Southeast - Troy Campus, Troy, USA; 4 Acute Care Surgery, Corewell Health Southeast - Troy Campus, Troy, USA

**Keywords:** minimally invasive splenectomy, actinomyces splenic abscess, campylobacter splenic abscess, campylobacter rectus splenic abscess, invasive campylobacter rectus, campylobacter rectus, splenic abscess

## Abstract

Splenic abscesses are rare pathologic conditions in which *Actinomyces* and *Campylobacter* species are rarely isolated. We present a 79-year-old female with multiple splenic abscesses from unknown sources with both *Actinomyces* species and *Campylobacter rectus* being isolated. She was initially treated conservatively with percutaneous drainage but eventually needed splenectomy. To our knowledge, this is the first documented case of *Campylobacter rectus* isolated from a splenic abscess.

## Introduction

Splenic abscesses are rare pathologic conditions in which *Actinomyces* and *Campylobacter* species are rarely isolated. The incidence of splenic abscesses is 0.14-0.70%. In the United States, Gram-positive bacteria, Enterobacterales and Anaerobes are the primary causative organisms [1]. Splenic abscesses are usually a result of bacteremia and occur more commonly in patients who are immunocompromised. Splenic abscesses are extremely morbid conditions with a mortality rate of up to 80% in immunocompromised patients with multilocular abscesses [2]. *Campylobacter rectus *(*C. rectus*) is found in the oral cavity and primarily causes infections confined to the oral cavity. Invasive infections caused by *C. rectus* are exceeding rare [3]. There are no prior cases that have documented *C. rectus* isolation from a splenic abscess. The gold standard described by many in the literature for the treatment of splenic abscesses is splenectomy, although percutaneous drainage is effective in certain situations [2]. Open splenectomy used to be the standard surgical approach; however, minimally invasive approaches are increasingly being performed due to their decreased morbidity, shorter hospital length of stay, and decreased pain encountered postoperatively [4].

## Case presentation

A 79-year-old female of Chinese descent, with a past medical history significant for a benign lung mass presented to the emergency room of a community hospital outside of Detroit, Michigan, United States, for 10 days of crampy left upper quadrant abdominal and flank pain. She had experienced some coughing 15 days prior that had since resolved. Associated symptoms included weakness, nonbloody and nonbilious emesis, decreased appetite, and significant weight loss. She denied experiencing diarrhea, hematochezia, or melena. She denied any recent travel, change in diet, or close family members with similar symptoms.

**Figure 1:**
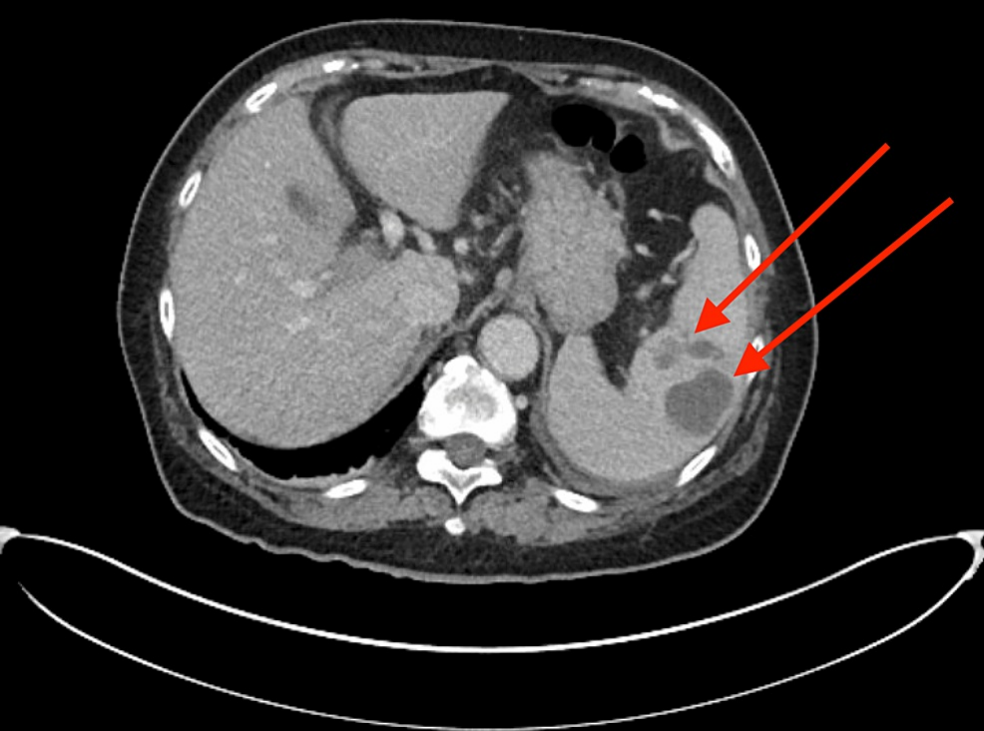
Axial CT view of multiple hypoenhancing lesions (red arrows) consistent with splenic abscesses on the patient’s admission.

Upon arrival, she had significant tenderness in the left upper quadrant and left flank. She was hemodynamically stable and afebrile with lab results showing a white blood cell count of 9.0 with a neutrophilic shift of 8.0 and a procalcitonin of 7.69. A CT with IV contrast of the abdomen and pelvis (Figure 1) revealed that the spleen had hypodense, heterogeneous fluid-filled lesions consistent with abscesses, without other abnormalities noted. The largest splenic fluid collection measured six centimeters in diameter. Urine cultures grew commensal flora, while blood cultures yielded no growth. Two-dimensional (2D) transthoracic echocardiography did not reveal any obvious vegetation.

The patient was admitted and started on IV vancomycin and piperacillin/tazobactam with general surgery and infectious disease consultations. The patient and family were offered surgical intervention vs. conservative management with the insertion of a percutaneous drain. Due to her age and overall functional status, they elected for percutaneous drainage. During drainage, approximately 40 cc of brown purulent fluid was aspirated with cultures growing *C. rectus*. Antibiotic coverage was transitioned to IV ampicillin/sulbactam. On hospital day four, the patient began experiencing chest pain and a CT of the chest showed a small effusion on the right, and a large- to moderate-sized effusion on the left. Due to concern for infective vs. reactive etiology, interventional radiology inserted a left chest tube the following day with laboratory evaluation showing no infectious process. The patient's abdominal pain persisted and a repeat CT abdomen/pelvis on hospital days five and eight showed resolution of the large abscess without change of the smaller abscesses.

Due to the continued failure to resolve her abdominal pain, on hospital day nine, the family elected for robotic-assisted laparoscopic splenectomy. During the operation, the splenic ligaments were ligated; however, upon further posterior mobilization, dense adhesions were encountered, especially near the percutaneous drain site. With the failure to fully mobilize the spleen, the decision was made to perform an open splenectomy via a left subcostal incision. Overall, she tolerated the procedure well with one unit of packed RBCs given intraoperatively due to blood loss, which was close to one liter. She received an additional unit later on after the operation due to tachycardia. Postoperatively, her pain was improved but she remained symptomatic from her recurrent left pleural effusion and on hospital day 13, she underwent video-assisted thoracoscopic surgery. She was discharged home on hospital day 20 on oral amoxicillin/clavulanic acid at which time her symptoms had resolved. Pathologic evaluation from the splenectomy later found *Actinomyces* on periodic acid-Schiff staining. On follow-up one week after discharge, she was doing well and was switched to a six-week course of IV ceftriaxone infusions due to the newfound *Actinomycosis* diagnosis. She will continue to follow up with the infectious disease clinic to monitor her clinical progression.

## Discussion

Lotfollahzadeh et al. performed a comprehensive review of splenic abscesses in 2018 and noted that splenic abscesses are uncommon presentations that are usually a result of bacteremia [2]. Chang noted the incidence of splenic abscesses as 0.14-0.70% [1]. Lotfollahzadeh et al. continued on to discuss that splenic abscesses have a bimodal age distribution with peaks in the third and sixth decades of life. They stated that approximately two-thirds of splenic abscesses in adults are solitary, and one-third are multiple in number. Splenic abscesses most commonly occur in immunocompromised patients or patients with endocarditis, neoplasia, trauma, splenic infarction, or diabetes. Immunocompromised patients with multilocular abscesses face an alarming mortality rate of up to 80%, whereas immunocompetent patients with unilocular abscesses have a comparatively lower mortality rate of 15%. Typical symptoms are fever and left upper quadrant pain. Physical findings are soft tissue edema, costovertebral tenderness, splenomegaly, left basilar rales, and dullness at the left lung base. Laboratory findings reveal a leukocytosis with a left shift. Lotfollahzadeh et al. stated that the gold standard for diagnosis is via CT scan [2]. Other radiographic imaging modalities such as bedside ultrasound can also be used to provide rapid diagnoses in certain settings of splenic abscesses as described by Chang [5].

Mustafa et al. described splenic abscesses as commonly isolating *Streptococcus* species, *Staphylococcus* species, *Mycobacterium*, parasites, and fungi. Uncommon causes are actinomycotic infections [6]. However, the causative organisms can vary by location as noted by Chang’s article notes in which *Burkholderia pseudomallei* was the most common cause in Kapit, Borneo, Malaysia [1]. The mainstay of the treatment involves parenteral antibiotics and source control of the infection either by splenic drainage or splenectomy as described by Lotfollahzadeh et al. When patients do not respond to treatment, the possibility of fungi, *Actinomyces*, or *Mycobacterium* as the underlying cause should be considered. Fungi, in particular, tend to respond well to antifungal treatment alone. Additionally, corticosteroid therapy has shown potential benefits in these patients [2]. Garduño et al. noted that *Actinomyces* are part of humans' typical intraoral flora makeup and are opportunistic organisms in nature that cause cervicofacial, thoracic, or abdominal infections. An immunocompromised state increases patients’ risk for actinomycotic infections with presumable mechanisms of entry via oral mucosal violation [7]. Jabr documented a rare case of actinomycotic splenic abscess after colonoscopy with polypectomy [8]. Chen et al. noted that actinomycotic detection is often difficult and requires tissue pathologic diagnosis as occurred in this case with *Actinomyces* detection only on pathologic tissue evaluation post splenectomy [9]. Garduño et al. recommend a treatment for actinomycotic splenic infections consisting of splenectomy in conjunction with four to eight weeks of parenteral penicillin. The regimen is then followed with four to eight weeks of oral penicillin [7].

Regarding *Campylobacter* infections resulting in splenic abscesses, cases in the literature are exceedingly rare. In 2014, Seng et al. described a case regarding an immunocompetent male who developed a single splenic abscess with *Campylobacter jejuni* after a case of febrile diarrhea in which his infection resolved with two percutaneous drainage tubes and a six-week course of ceftriaxone [10]. In 2022, Coustillères et al. describe a case of *Campylobacter fetus* found on blood culture in the setting of multiple splenic abscesses in an immunosuppressed renal transplant patient. This patient was found to have ileocolitis and was treated initially with IV ceftriaxone and oral metronidazole and discharged from the hospital. Five days later, the patient returned with abdominal pain, and multiple splenic abscesses were detected. She was treated with IV piperacillin/tazobactam and diflucan for two weeks, during which time, the splenic abscesses regressed and operative intervention was not employed. She did however suffer readmission later with diffuse systemic symptoms requiring further antibiotic intervention [11]. No cases of *C. rectus* isolated in splenic abscesses were discovered in the literature.

*C. rectus* is described by Lam et al. as an anaerobic inhabitant of the human oral flora commonly found in the periodontal sulcus, tongue, cheek mucosa, and saliva. It is associated with periodontal disease and is typically found in higher numbers in diseased subgingival sites compared to healthy ones. While *C. rectus* primarily causes localized disease in the oral cavity, there have been six previously reported cases of invasive *C. rectus* infections with one case being fatal. Interestingly, two of the affected patients were of Chinese descent [3]. In this patient’s case, she did not recall any recent periodontal infection or recent dental instrumentation. The mechanism of the bacteria occurring in the spleen is thought to be via introduction to the bloodstream. The most likely access sites would be via the oral cavity or other areas of the gastrointestinal tract, although she appeared asymptomatic from the primary infection. Another possibility is via an introduction from a left pleural effusion; however, the effusion appeared reactive in nature rather than causative. Regardless of the mechanism, this is the first documented case of splenic abscess with speciation of *C. rectus*. Not surprisingly, there is no set guideline or antibiotic regimen for treating splenic abscesses caused by *C. rectus;* however, Lim et al. reported that *C. rectus* is susceptible to amoxicillin-clavulanate, cefoxitin, clindamycin, imipenem, levofloxacin, and metronidazole [3].

In regard to surgical management of splenic abscesses, Lotfollahzadeh et al. stated that the gold standard treatment for a splenic abscess is splenectomy. However, recent studies have indicated successful alternative approaches based on the characteristics of the abscess. In high-risk patients or as a temporary solution before surgery, percutaneous aspiration offers a less invasive option, effectively bridging the gap to surgery and reducing the risk of a severe and potentially life-threatening infection. Percutaneous aspiration is particularly effective when the abscess collection is unilocular or bilocular, exhibiting a complete and thick wall without internal septations. The procedure is facilitated when the content of the abscess has low viscosity. In cases involving multiple collections or coagulopathy, laparoscopic or open surgical treatment is preferred over percutaneous drainage. Percutaneous drainage is less likely to be successful in the presence of multiple small abscesses, cavities filled with debris, coagulopathy, poorly defined cavities, diffuse ascites, and challenging access [2]. Chang stated that cases of splenic abscess caused by *Burkholderia pseudomallei* can be effectively treated with antibiotics alone [1].

When conventional therapy has failed or is contraindicated, splenectomy is the most viable option. Minimally invasive intervention is becoming increasingly utilized over open intervention as minimally invasive approaches yield decreased morbidity for the patient, shorter hospital length of stay, and decreased pain encountered postoperatively. Gamme et. al. described minimally invasive splenectomy as effectively treating a variety of splenic conditions, including splenic abscesses. There are several approaches described in the literature including multiport laparoscopic splenectomy, hand-assisted laparoscopic splenectomy, robotic-assisted laparoscopic splenectomy, single port laparoscopic splenectomy, and natural orifice laparoscopic surgery. Laparoscopic intervention has shown faster recovery times and fewer postoperative complications than traditional open techniques. Multiport laparoscopic splenectomy was considered the gold standard by the European Association of Endoscopic Surgery. Robotic splenectomy has not shown any clear advantage over other laparoscopic techniques. Gamme et. al. suggest that a hand-assisted laparoscopic splenectomy is an option for technically challenging splenectomies [4]. Regarding this case, the patient and family elected for conservative management initially; however, the patient clinically decompensated with worsening abdominal pain and dyspnea secondary to a large left pleural effusion. A splenectomy was then performed and the patient progressed well postoperatively. 

## Conclusions

Based on an extensive literature review, this is the first case of *C. rectus* isolated in a splenic abscess. Typically, *C. rectus* is a benign commensal bacteria associated with periodontal disease and is rarely associated with invasive infections. It was isolated in conjunction with *Actinomyces* species that were found as well, which are also a rare cause of splenic abscess. Overall, splenic abscesses are uncommon, morbid disease processes that usually affect immunocompromised individuals. These abscesses require coordination between medical and surgical teams for definitive management. Splenectomy is the gold standard for the treatment of splenic abscesses with minimally invasive techniques becoming more frequently utilized over traditional open surgical approaches. In this case, the patient was initially treated with antibiotics and percutaneous drainage per patient preference; however, she ultimately required operative intervention. Minimally invasive intervention was attempted, but due to scar tissue encountered during the procedure, a conversion to open splenectomy was necessary. Minimally invasive splenectomy is safe; however, a delay to definitive surgical intervention can make such procedures more technically challenging with the need for conversion to open surgical approaches.
